# Safety of liposome extended-release bupivacaine for postoperative pain control

**DOI:** 10.3389/fphar.2014.00090

**Published:** 2014-04-30

**Authors:** Juan Portillo, Nawal Kamar, Somayah Melibary, Eduardo Quevedo, Sergio Bergese

**Affiliations:** ^1^Department of Anesthesiology, Wexner Medical Center, The Ohio State UniversityColumbus, OH, USA; ^2^Department of Neurological Surgery, Wexner Medical Center, The Ohio State UniversityColumbus, OH, USA

**Keywords:** depofoam, bupivacaine, liposome bupivacaine, bupivacaine HCl, exparel

## Abstract

**Background:** Ideal postoperative pain management requires a multidisciplinary approach in combination with a variety of dosage regimens. Approximately 21–30% of patients experience moderate to severe pain in the postoperative period, which may have a significant impact on recovery rate, standard of living, psychological health, and postoperative complications.

**Objective:** Analysis of the incidence and characterization of reported adverse effects with DepoFoam bupivacaine compared to conventional bupivacaine or placebo.

**Methods:** A systematic review of prospective studies on the use of DepoFoam versus bupivacaine or placebo was performed in order to answer the clinically relevant question: is DepoFoam a safer formulation in place of bupivacaine single injection or continuous local infusion techniques for postoperative pain management? Inclusion criteria required randomized, controlled, double-blind trials in patients 18 years old or older, single dose used for postoperative pain control, and a primary procedure performed.

**Results:** Six studies fitted the inclusion criteria for analysis, DepoFoam bupivacaine used in therapeutic doses was well-tolerated, had a higher safety margin, and showed a favorable safety profile compared to bupivacaine and control groups.

**Conclusion:** Extended drug delivery system DepoFoam bupivacaine is a promising drug formulation that may significantly improve postoperative care and pain control in surgical patients.

## INTRODUCTION

An estimated 70 million surgical procedures are being performed annually in the United States (Cullen et al., 2009; Hall et al., 2010). Approximately 21–30% of these patients experience moderate to severe pain in the postoperative period, which may have a significant impact on their recovery, standard of living, psychological health, and the rate of postoperative complications. Additionally, healthcare costs of chronic pain developing from acute pain may constitute an enormous financial burden for both the patient and the community. The lifetime economic costs of a single 30-year-old patient suffering from chronic pain will reach one million U.S. dollars (Apfelbaum et al., 2003).

An ideal postoperative pain management requires a multi-modal and multi-disciplinary approach with a combination of various therapeutic maneuvers, acting at multiple levels of the nervous system and interfering with different pain propagation and perception mechanisms. Such a strategy will improve the overall results, ensure a higher level of postoperative analgesia, and reduce the possibility of side-effects including nausea, vomiting, constipation, and respiratory depression (Kehlet and Dahl, 2003). Additionally, an effective pain management control will shorten the inpatient time, reduce the rates of postsurgical complications and readmissions, prevent opioid dependence and, potentially, reduce mortality (White and Kehlet, 2010).

Currently adopted treatment protocols include various combinations of systemically or epidurally administered opioids, non-steroidal anti-inflammatory drugs, peripheral and neuraxial blocks and when indicated, supplementation with antidepressants, *N*-methyl D-aspartate (NMDA) antagonists and other centrally acting medications (Kehlet and Dahl, 2003; Bergese et al., 2011; Nigam et al., 2011).

Regional and local anesthesia plays an important role in postoperative pain control. In addition to effective analgesia, local anesthetics block the afferent neural stimuli from the surgical area, reducing endocrine-metabolic responses without any effects on inflammation (Kehlet and Dahl, 2003). Long-acting amide local anesthetics such as ropivacaine and bupivacaine provide a superior pain control over opioids and are commonly used in the postoperative period for infiltrative, regional, and neuraxial blocks (Ersayli et al., 2006; Kuthiala and Chaudhary, 2011; Sakai et al., 2013). Local anesthetics exert their effects via blocking the voltage-gated sodium channels on the cell membrane, thereby interfering with afferent signal propagation, thus reducing hyperalgesia and allodynia (Lai et al., 2004). Mechanically, some of these actions are achieved by a variable degree of blocking the potassium (K^+^), calcium (Ca^2^^+^) channels, and NMDA receptors.

Bupivacaine hydrochloride (HCl) is a widely used local anesthetic with prolonged duration of action. The drug is administered for local postoperative pain control either as a single bolus injection or continued infusion. In a study done by Borgeat et al. (2003) the average duration of interscalene block after a bolus injection varied between 8 to 12 h with either bupivacaine 0.5% or ropivacaine 0.5 or 0.75%. Apparently, the single bolus injection technique is not a sufficient method for postoperative pain management in most cases. On the other hand, placement and maintenance of perineural catheters for extended pain control will require additional training and skills for the clinicians, will be more costly, and will have a higher rate of complications (Chahar and Cummings, 2012). Thus, the development of novel, longer-acting local anesthetic formulations, like liposomal bupivacaine, is important to improve the management of postoperative pain (Chahar and Cummings, 2012).

A newer, extended-release formulation of bupivacaine, DepoFoam bupivacaine (EXPAREL, Pacira Pharmaceuticals, Inc., Parsippany, NJ, USA), was approved in the U.S. by the Federal Drug Administration (FDA) in October 2011. This innovative delivery system consists of biocompatible, biodegradable, spherical, lipid-based particles ranging in size between 10–30 μm and containing encapsulated drug designed to allow diffusion over an extended duration of time (Bergese et al., 2011; Chahar and Cummings, 2012). The encapsulated bupivacaine has a slower elimination half-life due to its slow release from the vehicle and decreased absorption rate from the injection site, resulting in sustained local analgesia (Bergese et al., 2011; Chahar and Cummings, 2012). The bupivacaine DepoFoam formulation has a bimodal plasma concentration time profile with an initial peak at 0.25–2 h and a second peak at 12–24 h after injection (Bergese et al., 2011). The liposomal formulation of bupivacaine is intended for a single-dose infiltration into the surgical site for prolonged postsurgical analgesia lasting up to 72 h (About DepoFoam, 2011). DepoFoam bupivacaine is mainly metabolized via hepatic conjugation and N-dealkylation of bupivacaine (Chahar and Cummings, 2012). Although, the bupivacaine and pipecolylxylidine (metabolite) concentrations are higher in patients with moderate hepatic impairment compared to patients with normal hepatic function, the differences are not clinically significant and do not require any dose adjustments according to the FDA guidelines (Chahar and Cummings, 2012).

### SAFETY PROFILE

The most significant adverse effects (AEs) of bupivacaine are primarily related to its cardiovascular and neurotoxic properties (Pacella et al., 2010). The drug is 6 to 10 times more cardiotoxic than lidocaine, an effect attributed to the blockade of sodium, potassium, and L-type calcium channels in the sarcolemma of cardiomyocytes that produce a conduction block, reduce contractility, depress automaticity (spontaneous phase IV depolarization), and reduce the duration of the refractory period (Kiuchi et al., 2011; Chahar and Cummings, 2012). Bupivacaine-induced cardiac arrests are more resistant to resuscitative interventions compared to other local anesthetics (Beilin and Halpern, 2010). Bupivacaine can also induce Ca^2^^+^-induced apoptosis of muscle cells, a complication described after performing retrobulbar and peribulbar blocks (Chahar and Cummings, 2012).

An important question arises as to whether or not this liposomal delivery system will lead to fewer AEs and have a better safety profile when compared to a placebo group or a conventional bupivacaine formulation. Despite experimental evidence (Richard et al., 2011), there have been no reports in the current literature of post-injection granuloma formation in humans.

A comprehensive review on the pharmacological characteristics of the sustained-release liposome formulation DepoFoam has become recently available (Chahar and Cummings, 2012). Nevertheless, from a clinician’s point of view, it would be extremely valuable to summarize the existing limited literature reports on DepoFoam bupivacaine’s efficacy, tolerability, and safety profile.

## METHODS

We reviewed published prospective studies within the last 5 years on head to head comparison of DepoFoam versus bupivacaine or placebo to answer the clinically relevant question whether DepoFoam would be a safer formulation capable of replacing bupivacaine single injection or continuous local infusion techniques in the postoperative pain management.

We also conducted a systematic review of published, randomized controlled trials that detailed the AEs of DepoFoam in comparison to a placebo group or a conventional bupivacaine formulation.

Two individual investigators conducted separate literature searches to isolate clinical trials that reported on the AEs of DepoFoam bupivacaine in the postoperative period. Using the preferred reporting items for systematic reviews and meta-analysis (PRISMA) guidelines, a search in PubMed, Scopus, and the Cochrane Library was performed in March of 2013 with the following keywords: *DepoFoam, bupivacaine, liposome bupivacaine, bupivacaine HCl,* and *Exparel*.

The following inclusion criteria were used:

(1) Randomized controlled double blind trials in patients aged 18 or older.
(2) Use of a single dose of DepoFoam bupivacaine for postoperative pain control.
(3) A primary procedure was performed (i.e., not a salvage or follow-up procedure).

The objective of this analysis was to identify the most common reported AEs experienced with escalating doses of DepoFoam bupivacaine when compared to conventional bupivacaine or placebo. A sub-analysis of the most common AEs related to treatment was also conducted and interpreted.

## RESULTS

A total of 16 articles pertaining to DepoFoam bupivacaine were identified through the initial search. However, only six studies fitted the inclusion criteria for analysis. These studies were conducted based on proper obtainment of written informed consent, strict adherence to the amendments of the Declaration of Helsinki, the Good Clinical Practices guidelines, and approval of the Institutional Review Board (IRB). The types of surgeries encompassed were total knee arthroplasty (Bramlett et al., 2012), bunionectomy (Golf et al., 2011), hemorrhoidectomy (Gorfine et al., 2011), breast augmentation (Smoot et al., 2012; Minkowitz et al., 2012), and a study conducted on healthy volunteers (Naseem et al., 2012). The AEs were reported as deemed appropriate by the investigator and the relationship to study drug administration was categorized as clinical or non-clinical significant.

The randomization groups and demographic data for the six studies are outlined in **Table 1**.

**Table 1:** Randomization groups and demographic characteristics.

Study	Dose	*n*	Mean age and standard deviation (SD)	Males	Females	Surgery
Bramlett et al. (2012)	DepoFoam 133 mg	27	61.4 (7.0)	53	85	Knee arthroplasty
*n* = 138	DepoFoam 266 mg	25	61.1 (8.7)
^*^134 patients completed the study.	DepoFoam 399 mg	26	61.8 (6.3)
	DepoFoam 532 mg	24	64.9 (7.3)
	Bupivacaine HCl 150 mg	32	62.2 (7.2)
Golf et al. (2011)	DepoFoam 120 mg	93	42.4 (12.7)	34	159	Bunionectomy
*n* = 193	Placebo	92	43.3 (13.4)
^*^185 patients completed the study.			
Gorfine et al. (2011)	DepoFoam 300 mg	95	48.0 (12.2)	130	59	Hemmorrhoidectomy
*n* = 189	Placebo	94	48.7 (11.9)
^*^186 patients completed the study.		
Minkowitz et al. (2012)	DepoFoam 150 mg + bupivacaine HCl 75 mg	17	32.2 (7.2)	N/A	17	Augmentation mammoplasty
*n* = 94	DepoFoam 266 mg + bupivacaine HCl 75 mg	14	29.3 (6.3)		14
^*^94 patients completed the study.	DepoFoam 532 mg	31	32.9 (7.6)		31	
	Bupivacaine HCl 200 mg	32	30.8 (7.1)		32	
Naseem et al. (2012)	Varying doses	49	26 (5)	34	15	Healthy volunteers
*n* = 49	
^*^46 patients completed part 1 of the study.	
^*^16 patients completed part 2 of the study.	
Smoot et al. (2012)	DepoFoam 600 mg	64	30.8 (7.3)	N/A	134	Augmentation mammoplasty
*n* = 136	Bupivacaine HCl 200 mg	70	30.6 (7.6)
^*^82 patients completed the study.	

*Total: *n* = 743*

* The actual number of patients who completed the study which was different from the n number.

Bramlett et al. (2012) conducted a randomized, double-blind, dose-ranging study of DepoFoam bupivacaine compared to bupivacaine HCl in a phase 2 trial of 138 patients undergoing total knee arthroplasty. All the doses of DepoFoam bupivacaine and bupivacaine HCl were reported to be well tolerated and safe. No significant AEs were attributed to the study drug, and the incidence rate of AEs for bupivacaine DepoFoam were similar to those found for bupivacaine HCl.

Golf et al. (2011) conducted a phase 3, randomized, placebo-controlled, double-blind study of DepoFoam bupivacaine in 193 bunionectomy patients. The study authors concluded that DepoFoam bupivacaine 120 mg was well tolerated and safe and it provided a higher level of analgesia. Analgesia level was assessed using the area under the curve (AUC) for the pain numeric rating scale during 24 h, the pain-free period, and the use of opioids. The incidence of medication-related AEs and severe adverse events (SAE) were higher for the DepoFoam bupivacaine group (9.3 versus 5.2 % and 11.3 versus 5.2%, respectively). However, the incidence of moderate AEs was found to be higher for the placebo group. In fact, the only AE that had a significantly higher occurrence rate in the DepoFoam group was postoperative nausea and vomiting (PONV), a well-known side effect of bupivacaine injection. Because the study was designed to compare DepoFoam with placebo, no conclusions could be made regarding the incidence of PONV in the DepoFoam bupivacaine group versus conventional bupivacaine group. Above all, the AEs were considered to be unrelated to therapy, and no statistically significant changes were found in blood tests, except for two cases of elevated creatinine levels in the DepoFoam group.

Gorfine et al. (2011) conducted a phase 3 randomized, double-blind, placebo-controlled trial of 300 mg DepoFoam bupivacaine in 189 patients undergoing hemorrhoidectomy. The cumulative pain score was assessed during the first 72 h after study drug administration using the AUC of pain intensity. The incidence and amount of opioid rescue medication used, time to initial use of a rescue drug, and the level of patient satisfaction were also measured (Gorfine et al., 2011). Use of DepoFoam bupivacaine resulted in a statistically significant improvement in all the above mentioned parameters when compared with placebo.

This study found that administration of DepoFoam bupivacaine at a dose of 300 mg was safe and well tolerated in all patients. The only AE attributed to the study drug was a single case of fingernail redness that resolved on day 6 without any intervention.

Minkowitz et al. (2012) reported on the clinical sequelae noted on 94 female patients who had undergone augmentation mammoplasty, during a 2-year follow-up period. The paper included two studies: a phase II, active-controlled, randomized, double-blind, multi-center study, and a phase III, randomized, double-blind, multi-center study. Both studies reported that the DepoFoam bupivacaine was well tolerated and no SAEs were reported. Moreover, the AEs of mild or moderate intensity were not deemed to be related to the study medication. Overall, the study found no significant tolerability difference between the two arms of either study. The authors concluded that administration of the DepoFoam bupivacaine would not have a negative effect on the integrity of breast implants at 2 years after the surgery.

Naseem et al. (2012) characterized the effects of subcutaneous administration of DepoFoam bupivacaine of varying doses on the QTc interval in 49 healthy volunteers. The study consisted of two parts: part 1 was a single-center, randomized, placebo- and positive-controlled, double-blind, double-dummy, and crossover study that involved a sequence of four treatments (400 mg moxifloxacin, 300 mg DepoFoam bupivacaine, 450 mg DepoFoam bupivacaine, and a placebo group), whereas part 2 was a single-center, sequential-dose, exploratory extension to test a higher dose of DepoFoam that involved a sequence of three treatments (600 mg DepoFoam bupivacaine, 750 mg DepoFoam bupivacaine, and a placebo).

In the first part of the study, all doses of DepoFoam were reported to be well tolerated by the healthy participants. One SAE (acute hepatitis A with eosinophilia) was reported in the 450 mg DepoFoam formulation group that was deemed unrelated to DepoFoam treatment. Sixty (60) AEs were reported by 25 participants in the part 1 study, of which 17 were considered treatment-related (pooled data from DepoFoam bupivacaine and moxifloxacin treatment groups). The most common AEs experienced in the DepoFoam bupivacaine group included abdominal pain, injection site irritation, headaches, nervousness, gastrointestinal, and respiratory/thoracic symptoms. In part 2, no SAEs were reported. A total of 43 AEs were reported by ten patients, of whom eight were deemed to be treatment-related (pooled data from DepoFoam and moxifloxacin treatment groups). The most common AEs reported by participants in the second part of the study were injection site erythema, pruritus, injury, poisoning, nervous system, and gastrointestinal disorders, with no additional information provided by the authors. In relation to our objective, all four doses of DepoFoam bupivacaine across both studies failed to show any clinically significant effects on the QTc interval, leading the researchers to conclude that doses up to 750 mg ofDepoFoam bupivacaine pose no cardiac concern, with no QT interval prolongation.

Smoot et al. (2012) conducted a randomized, active-controlled, multi-center, double-blind study of 136 female patients undergoing bilateral augmentation mammoplasty. This was a double-arm study comparing DepoFoam bupivacaine 600 mg to conventional bupivacaine HCl 200 mg. There were no SAEs reported for either arm. A total of 99 patients (72.8%), with 48 patients in DepoFoam bupivacaine group and 51 patients in the bupivacaine HCl group, reported at least one AE. The majority of AEs were mild or moderate in severity. Given the comparable safety profiles of the two administrations, and the lack of treatment-related serious AEs, the study concluded that DepoFoam bupivacaine was well tolerated in patients undergoing augmentation mammoplasty, even though the study was underpowered to achieve statistical significance.

**FIGURE 1:**
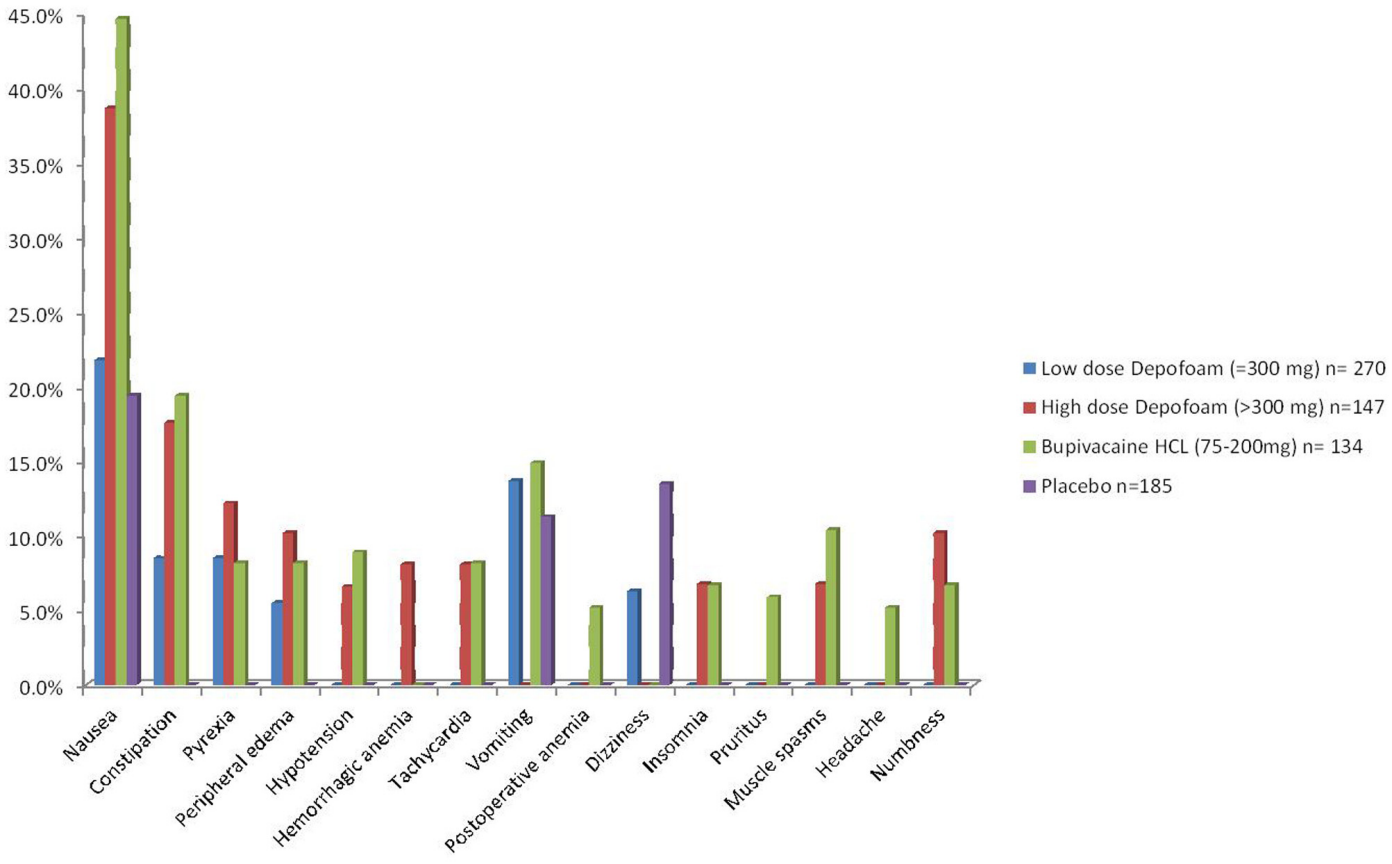
**Most common adverse effects reported (incidence above 5.0%)**.

A summary of the most common AEs reported (above 5.0% incidence) in the trials are outlined in **Figure 1**.

## DISCUSSION

The balance between drug efficacy and safety remains at the forefront of debate regarding postsurgical pain management. Current clinical practices include the use of opioid and non-opioid analgesics along with various regional anesthesia techniques combined with alternative methods and medications, when indicated. Local anesthetics and bupivacaine in particular, are known to cause serious cardio- and neurotoxic side effects (Srinvivasa et al., 2003; Yang et al., 2011; Bergese et al., 2012; Oda and Ikeda, 2013). The introduction of the liposome extended-release bupivacaine formulation used for the postoperative pain control provides a new and promising method, leading to significant changes in patient management. Current experimental data suggest that lipid emulsion-based bupivacaine solutions have a higher safety margin compared to conventional bupivacaine. Oda and Ikeda (2013) showed through an *in vivo* model that higher doses (above 300 mg) of lipid bupivacaine are required to induce convulsions (neurotoxicity) and cardiac arrest.

This review summarizes the results reported in six studies on the safety profile of DepoFoam bupivacaine used in infiltrative anesthesia for postoperative pain control. In these reports, the authors compared DepoFoam bupivacaine with conventional bupivacaine solution, and control medication or placebo. In general, the findings indicated that DepoFoam bupivacaine used in therapeutic doses was well-tolerated, and showed a favorable safety profile compared to bupivacaine and controls. Golf et al. (2011) reported two cases of blood creatinine elevation in the DepoFoam group. Unfortunately, the authors did not describe whether these changes were related to drug use or other medical conditions (Golf et al., 2011). Additionally, Gorfine et al. (2011) reported a patient in the DepoFoam bupivacaine group, who experienced finger nail redness on day 2, which was considered by the investigator to be related to the study drug.

For future studies, a better-powered double-blind prospective trial with a higher number of patients will be required to address the questions regarding the actual incidence rate of AEs and their severity. Additionally, future studies should focus on drug use during different surgical procedures, targeting patients with various comorbidities, particularly kidney dysfunction and dyslipidemia. It would be clinically relevant to assess the incidence of PONV in randomized patient groups undergoing various surgical interventions and receiving bupivacaine or DepoFoam bupivacaine for postoperative pain control. It will also be important to take into account the relatively large size of the lipid multivesicular liposomes (10–30 μm) which lengthens the time of local anesthetic action by slowing release from the liposome, furthermore, delaying the peak plasma concentration (Chahar and Cummings, 2012). Additionally, the effects of DepoFoam bupivacaine on the blood lipid profile and the development of hemorrhagic anemia with the administration of high-dose DepoFoam is a side effect worth further evaluation, whether this may be a drug-induced vasculitis, thrombophlebitis, or a possible lipid microembolism.

In conclusion, extended drug delivery system of DepoFoam bupivacaine is a promising drug formulation which may significantly improve the postoperative pain control in surgical patients. Further studies with larger patient groups are needed to enhance the current level of knowledge of the drug’s advantages and disadvantages, and define the areas of best application.

## Conflict of Interest Statement

The authors declare that the research was conducted in the absence of any commercial or financial relationships that could be construed as a potential conflict of interest.
